# Quantitative IC_50_ Analysis of Puromycin-Induced Cytotoxicity in NIH/3T3 Cells Using a Multi-Well Array Impedance Biosensor

**DOI:** 10.3390/bios15090572

**Published:** 2025-09-01

**Authors:** Seok-kyu Kim, SuGwon Nam, Moongyu Jang

**Affiliations:** 1 School of Nano Convergence Technology, Hallym University, Chuncheon 24252, Republic of Korea; hakillike98@naver.com (S.-k.K.); sugwonnam@gmail.com (S.N.); 2 School of Semiconductor & Display Technology, Hallym University, Chuncheon 24252, Republic of Korea; 3 Center of Nano Convergence Technology, Hallym University, Chuncheon 24252, Republic of Korea

**Keywords:** semiconductor, NIH 3T3 cell, IC_50_, ECIS, puromycin, capacitance

## Abstract

ECIS-based impedance biosensors have been extensively studied in various fields including cancer research, microbiology, and immunology. However, most studies have primarily focused on monitoring cellular behavior through impedance changes, with relatively less emphasis on interpreting the biological significance of impedance signals. In this study, we employed a multi-well array impedance biosensor to conduct IC_50_ (half-maximal inhibitory concentration) analysis, a widely used metric for evaluating drug efficacy and toxicity in biological and pharmacological research. Specifically, we assessed the IC_50_ values of puromycin, an aminonucleoside antibiotic known to inhibit protein synthesis. NIH/3T3 fibroblasts were exposed to various concentrations of puromycin, and real-time impedance monitoring was performed. Cell viability was assessed, and the IC_50_ value of puromycin for NIH/3T3 cells was determined to be 3.96 µM using capacitance-based impedance analysis. Our findings demonstrate that the multi-well array impedance biosensor provides a rapid and quantitative method for drug toxicity evaluation, offering a valuable platform for drug screening and biocompatibility assessment.

## 1. Introduction

Biosensors have emerged as essential tools in modern drug development, offering real-time monitoring capabilities for evaluating drug efficacy and toxicity [1]. The process of discovering and optimizing new drugs is inherently complex and time-consuming, requiring efficient analytical techniques for assessing cellular responses to therapeutic compounds [2]. Traditional methods for drug evaluation typically involve analyzing cell viability, proliferation, morphology, and metabolic activity as well as detecting intracellular biomarkers [3,4]. However, these techniques often rely on labeling or endpoint assays, which can be invasive and fail to capture dynamic cellular responses [5].

To address these limitations, electric cell-substrate impedance sensing (ECIS) has been widely adopted as a label-free, non-invasive method for the real-time monitoring of cellular behavior and drug interactions. ECIS technology, originally developed by Giaever and Keese in 1984, quantifies changes in cellular adhesion, proliferation, and barrier integrity by measuring electrical impedance variations [6,7,8]. These impedance signals provide valuable insights into cell behavior under different experimental conditions, including exposure to cytotoxic compounds, making ECIS a powerful tool for cancer research, microbiology, and immunology [9,10,11].

Recent advancements in ECIS-based biosensors have focused on improving sensitivity, expanding analytical capabilities, and enabling high-throughput screening Considerable efforts are being made in the field of ECIS-based biosensors to establish and define the relationship between electrical signals indicated by impedance and the biological state of cells [12,13].

For instance, Ebrahim et al. (2022) [14] systematically applied electric cell-substrate impedance sensing (ECIS) to telomerase-immortalized corneal epithelial cells in order to rigorously define the quantitative relationship between impedance signals and cellular biological states. By measuring key electrical parameters including resistance (R), capacitance (C), junctional resistance (R_b_), and the α parameter associated with cell–substrate interactions—the study provided detailed insights into how variations in cellular barrier function, motility, and morphology manifest in the impedance spectra. Through comprehensive mathematical modeling and time–frequency analysis, the authors demonstrated that changes in cell structure, cell–cell adhesion, and migratory behavior can be accurately tracked and correlated with characteristic impedance signal, thereby underscoring the utility of ECIS as a powerful, label-free tool for the real-time monitoring of dynamic cellular processes.

Pradhan et al. (2021) [15] introduced a novel four-electrode ECIS-based biosensor to investigate the cytotoxic effects of tamoxifen on HeLa cervical cancer cells. By performing frequency-dependent impedance measurements across a broad range (100 Hz to 1 MHz), the study captured dose-dependent impedance reductions that reflected progressive cell detachment, morphological changes, and cell death. The authors constructed dose–response curves from the impedance data and determined IC_50_ values, which showed strong concordance with those obtained from conventional biochemical assays including MTT, live/dead staining, and flow cytometry. Their findings validate ECIS as a reliable, label-free, and real-time analytical platform for quantitative cytotoxicity assessment and precise IC_50_ determination.

A previous study developed a multi-well array impedance biosensor to facilitate the analysis of drug effects under various conditions. These biosensors allow for simultaneous testing of multiple drug concentrations or different therapeutic compounds, enabling researchers to efficiently compare cytotoxic effects and determine dose–response relationships. By minimizing non-specific interactions and providing independent testing environments within each well, multi-well ECIS systems offer an effective solution for drug screening and biocompatibility testing [16].

Despite the extensive research on ECIS biosensors, many studies have primarily focused on tracking impedance changes associated with cell behavior rather than interpreting the biological significance of these signals [17]. There remains a need for establishing clear relationships between impedance-based measurements and cellular responses to drugs such as apoptosis and necrosis. Understanding these correlations is critical for improving the accuracy of drug toxicity assessments and expanding the application of ECIS biosensors in pharmaceutical research [18,19].

Several commercial EIS-based systems, such as the xCELLigence RTCA (Roche/Agilent Technologies, Santa Clara, CA, USA) and the ECIS platform (Applied BioPhysics, Troy, NY, USA), have been widely applied for the real-time monitoring of cell viability, proliferation, and drug responses. While these systems provide reliable biological insights, they often suffer from limitations including high operational cost, proprietary instrumentation, and limited scalability. In contrast, the present study employs a semiconductor-fabricated multi-well array impedance biosensor that offers a cost-effective and reproducible alternative for quantitative drug toxicity assessment [20,21].

This study aimed to address these challenges by developing a multi-well array impedance biosensor capable of quantifying drug-induced cytotoxic effects through IC_50_ (half-maximal inhibitory concentration) analysis. Using this biosensor, we evaluated the cytotoxicity of puromycin, well-characterized inhibitors of protein synthesis. By applying mathematical modeling to impedance data, we aimed to provide a quantitative framework for analyzing drug effects on cell viability. Our findings contribute to the advancement of real-time drug screening technologies, demonstrating the potential of multi-well array ECIS biosensors as efficient platforms for evaluating drug toxicity and cellular responses.

## 2. Materials and Methods

### 2.1. Multi-Well Array Impedance Biosensor Fabrication Process

**Figure 1:**
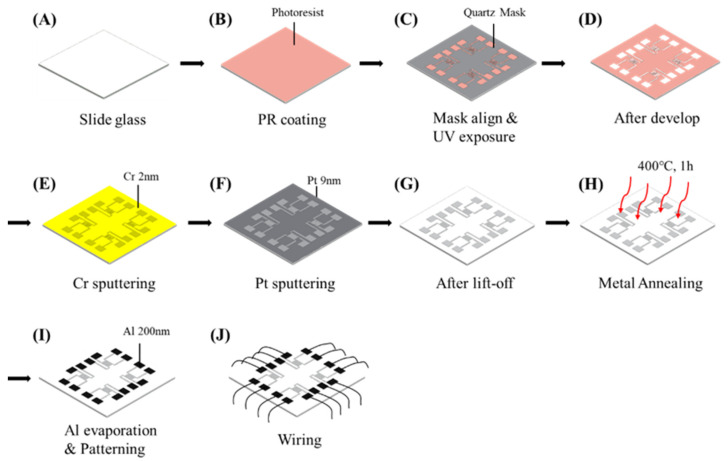
Fabrication steps of a multi-well array impedance biosensor based on semiconductor processing techniques. (**A**) glass substrate; (**B**) photoresist (PR) coating; (**C**) exposure; (**D**) development; (**E**) chromium (Cr) deposition; (**F**) platinum (Pt) deposition; (**G**) lift-off; (**H**) annealing; (**I**) pad deposition; (**J**) wire bonding.

Figure 1 illustrates the step-by-step fabrication process of a multi-well array impedance biosensor using a semiconductor-based manufacturing method. A 4 × 4 cm glass slide served as the substrate. Photoresist (PR) (AZ 5214E, MicroChemicals GmbH, Ulm, Germany) was applied to the entire surface of the substrate through spin coating using a CY-SP4 spin coater (Jungmun-Science, Seoul, Republic of Korea) at 4500 rpm for 35 s. To enhance adhesion between the PR and the substrate, a soft-bake process was conducted at 100 °C for 10 min on a hot plate (HPLP-A-P, Seoul, Republic of Korea).

Subsequently, a quartz mask with an array impedance pattern was aligned on the substrate, followed by i-line ultraviolet (UV) exposure (EVG 610, *λ* = 365 nm) for 10.4 s. The sample was then developed using AZ 300 MIF Developer (Merck KGaA, Darmstadt, Germany) for 40 s, and any residual developer was removed through deionized water rinsing for 1 min.

To deposit metal onto the pattern, radio frequency/direct current magnetron sputtering (SHS-2M3-40T, SamHan Vacuum Development, Paju-si, Republic of Korea) was employed. The deposited metals included platinum (Pt) and chromium (Cr), both with a purity of 99.95%, supplied by iNexus, Inc. Initially, a 2 nm layer of Cr was deposited to enhance the adhesion between the substrate and Pt. Following this, 9 nm of Pt, known for its high biocompatibility and work function of approximately 5.2–5.9 eV, was deposited.

After sputtering, a liftoff process was carried out to remove the residual PR by immersing the sample in acetone for 10 min while using an ultrasonic cleaner. To mitigate surface damage and stabilize increased resistance caused during fabrication, heat treatment was applied at 400 °C for 1 h in an argon environment using a SHTC-3000 furnace (SamHan Vacuum Development, Paju-si, Republic of Korea).

For wiring and measurement stability, a shadow mask was carefully aligned on the sample, and 200 nm of aluminum was deposited in the pad area via thermal evaporation (SHE-6T-350D, SamHan Vacuum Development, Paju-si, Republic of Korea) to protect the pad region. The final fabrication step involved connecting the impedance measurement device by wiring the sensor. Teflon-insulated wires with a 0.16-mm core and a 0.32-mm solid wire were used for this purpose.

### 2.2. Process for Creating the Cell Culture Environment

**Figure 2:**
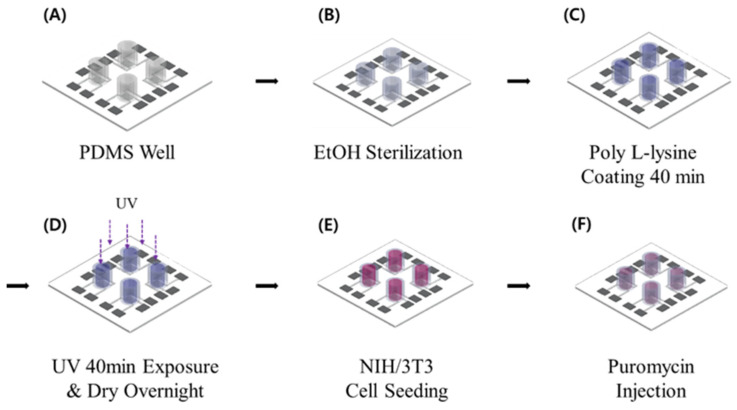
Process for establishing the internal environment of the well. (**A**) establishment of the cell culture environment; (**B**) sterilization process; (**C**) poly-L-lysine coating; (**D**) sterilization and drying; (**E**) cell seeding; (**F**) drug treatment.

**Figure 3:**
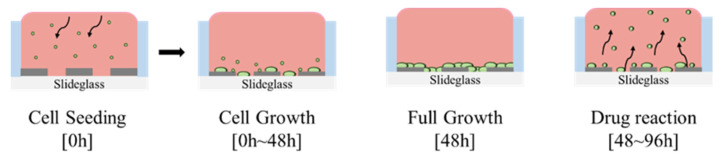
Illustrative representation of the cell seeding, proliferation, and drug response process.

**Figure 4:**
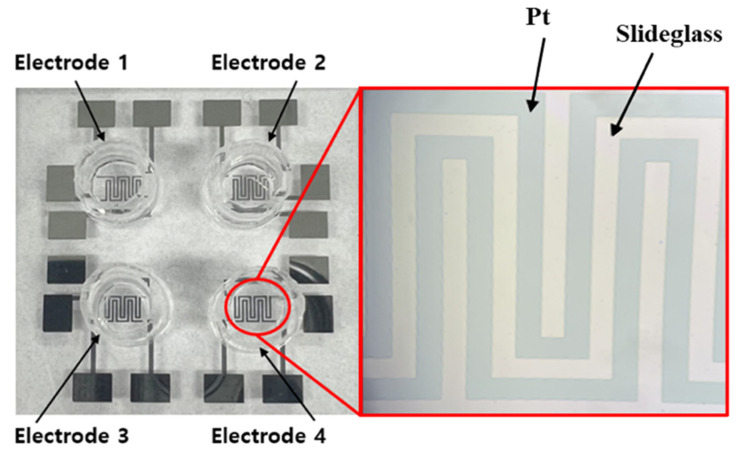
Micrograph of multi-wells 1 to 4 and the impedance pattern of the multi-well array impedance biosensor.

Figure 2 presents a schematic representation of the cell-seeding procedure. A multi-well structure, designed to create independent microenvironments, was fabricated using PDMS and integrated with the sensor pattern. The PDMS well was prepared using a biopsy punch with an inner diameter of 6 mm, an outer diameter of 8 mm, a height of 7 mm, and a total capacity of 198 μL. A 5:1 mixture of PDMS and hardener was used as an adhesive to ensure firm attachment of the well to the sensor surface. Once the PDMS well was attached, it underwent heat curing on a hotplate (HPLP-A-P) at 100 °C for 2 h to fully cure the adhesive and prevent leakage. To prepare the well for cell culture, phosphate-buffered saline (PBS) (Dulbecco™, Thermo Fisher Scientific, Waltham, MA, USA) was injected into the PDMS well for rinsing. The interior of the well was sterilized with 70% ethanol (EtOH) to eliminate potential contaminants before cell seeding. This sterilization cycle was repeated three times using PBS and 70% EtOH to ensure thorough decontamination. For enhanced cell adhesion, poly-L-lysine (0.01% purity, Sigma-Aldrich, St. Louis, MO, USA) was introduced into the well and incubated for 40 min, facilitating ionic bonding between the cells and the patterned sensor surface. During this process, UV irradiation was applied for sterilization. After 40 min, excess poly-L-lysine was removed, and the well was dried under UV light for 24 h. To minimize medium evaporation during the experiment, a separate PBS-containing well was placed adjacent to the main PDMS wells. Previous studies have indicated that the evaporation of small amounts of medium has a negligible effect on the impedance values [22]. Figure 3 illustrates this preparation process and the configuration of the PDMS well structure used for cell culture experiments. Following the completion of the cell culture environment setup, 5628 NIH/3T3 fibroblast cells, derived from mouse embryos, were seeded into the well and incubated in a CO_2_ incubator (Vision Scientific Corporation, VS-2050C, Vision Scientific Corporation, Daejeon, Republic of Korea) under controlled conditions of 5% CO_2_, 37 °C, and 95% humidity. Figure 4 shows actual images of the fabricated PDMS multi-well structure and platinum electrode array, illustrating the final sensor configuration used for cell culture and impedance measurements. 

**Figure 5:**
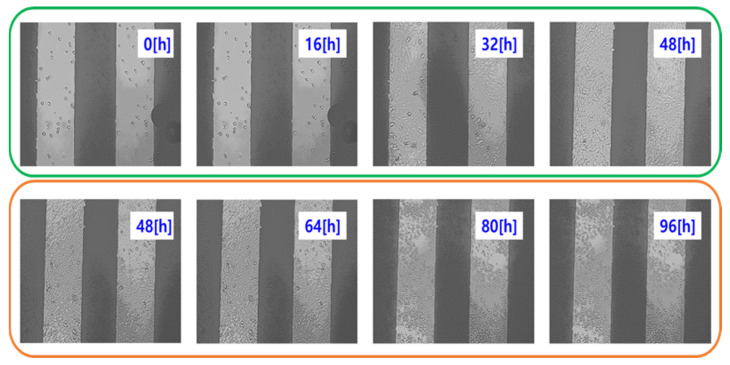
Real cell images measured with a real cell behavior analyzer (JuLi^TM^ Br&FL).

Figure 5 shows representative live-cell images of NIH/3T3 cells captured using the JuLi™ Br&FL live cell movie analyzer (NanoEnTek Inc., Seoul, Republic of Korea). These images confirm the correlation between the impedance-derived capacitance variations and actual cellular responses during attachment, proliferation, and cytotoxicity.

### 2.3. Cell Culture and Drug Preparation

NIH/3T3 fibroblast cells (mouse embryonic fibroblasts) were obtained from the Korean Cell Line Bank (Seoul, Republic of Korea). These cells, originally derived from mouse embryonic tissue, are widely used in studies of cell proliferation, adhesion, migration, and oncogenic transformation due to their stable growth characteristics and responsiveness to growth factors and cytotoxic agents. The NIH/3T3 fibroblasts were cultured in Dulbecco’s modified Eagle’s medium (DMEM; Gibco™, Thermo Fisher Scientific, Waltham, MA, USA) supplemented with 10% fetal bovine serum (FBS; Gibco™) and 1% antibiotic-antimycotic solution (Gibco™). The cells were maintained at 37 °C in a humidified atmosphere containing 5% CO_2_ and 95% relative humidity using a CO_2_ incubator (VS-2050C, Vision Scientific Corp., Daejeon, Republic of Korea). The culture medium was refreshed every two to three days, and cells were passaged upon reaching approximately 80–90% confluence.

Puromycin (CAS No. 58-58-2; ≥98% purity) was purchased from Sigma-Aldrich (St. Louis, MO, USA). Working concentrations were freshly prepared by diluting the stock solution in culture medium immediately prior to each experiment. Puromycin is an aminonucleoside antibiotic that inhibits protein synthesis by mimicking aminoacyl-tRNA and causing the premature termination of translation, ultimately leading to cell death. In biosensor applications, particularly ECIS-based studies, puromycin is frequently used as a model cytotoxic agent to induce cell detachment, loss of barrier function, and cell lysis. Its well-characterized mechanism of action and predictable impedance signature make it a standard agent for validating the sensitivity and dynamic range of impedance-based cytotoxicity assay determinations.

### 2.4. Impedance-Based Measurement of Cell Viability

**Figure 6:**
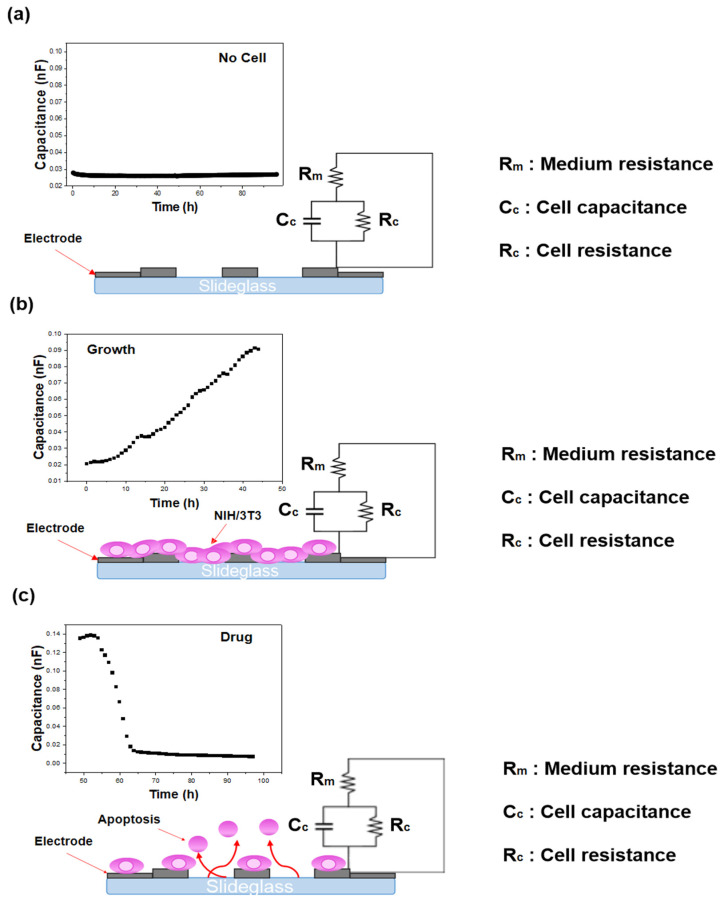
ECIS measurement principle and circuit diagram: (**a**) no cells, (**b**) cell growth process, and (**c**) cell death process.

Cell behavior was monitored using multi-well array impedance biosensors, which detect variations in impedance. Measurements were performed using a Keithley Agilent 4284A (Keysight Technologies, Santa Rosa, CA, USA) impedance analyzer over a 96-h period, with frequency sweeps ranging from 1 kHz to 1 MHz and an excitation voltage of 0.1 V. Although impedance responses across the full frequency range (1 kHz–1 MHz) can provide additional information, this aspect was investigated in detail in our previous study [16,22]. To avoid redundancy, the frequency sweep data are not re-presented here. Instead, the present work focuses on the capacitance response at 250 kHz, the frequency identified in our earlier analysis as the most reliable indicator of cellular behavior with high reproducibility. The integration time was configured to the ‘mid’ setting (short, mid, long). A schematic illustration of the impedance principle of measurement is shown in Figure 6. To verify that the impedance changes corresponded to actual cellular activity, cell images were captured concurrently using a live cell movie analyzer (JuLi™ Br&FL, NanoEnTek, Waltham, MA, USA). Previous studies have confirmed the reproducibility and reliability of this biosensing system for cell and drug evaluation through Trypan blue exclusion and Cell Counting Kit-8 (CCK-8, (Gibco™, Thermo Fisher Scientific, Waltham, MA, USA)) assays [23]. In this work, the capacitance component of the impedance signal was emphasized, as it enables the intuitive detection of cell state changes including growth, death, and biological responses [24]. In addition, to exclude possible artifacts from medium evaporation during the 96-h monitoring period, we referred to our previous investigation, which demonstrated that evaporation effects were negligible under similar experimental conditions [22].

## 3. Results

### 3.1. IC_50_ Determination of Puromycin in NIH/3T3 Cells

**Figure 7:**
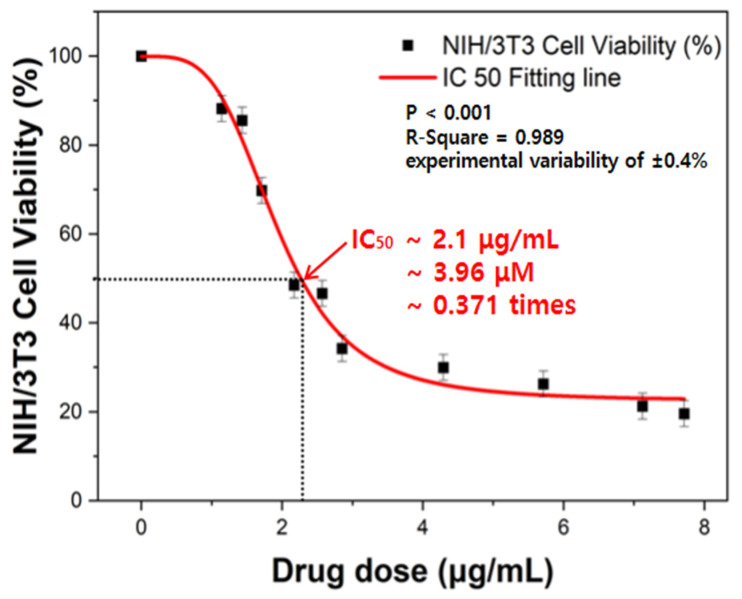
Results of the IC_50_ analysis for NIH/3T3 cell viability (250 kHz).

To evaluate the cytotoxic effects of puromycin on NIH/3T3 fibroblasts, the cells were treated with varying drug concentrations ranging from 0.2 times to 1.35 times the standard dose, and real-time impedance monitoring was conducted over 96 h. Figure 7 shows the results of the IC_50_ analysis. The IC_50_ value refers to the drug concentration required to inhibit 50% of the cell’s biological activity, indicating the puromycin concentration at which the NIH/3T3 cell viability is reduced to 50%.

The dose–response relationship of NIH/3T3 cells treated with puromycin was analyzed using a four-parameter logistic (4PL) model, which is widely employed for pharmacological IC_50_ determination. The equation used for IC_50_ analysis is presented in Equation (1):

(1) $$Y=C_{0}+\;(C_{M}-C_{0})/1+{\left(X/{IC}_{50}\right)}^{HillSlope}$$

where *C*_0_ and *C_M_* denote the minimum and maximum asymptotic responses, respectively; *X* represents the drug concentration; and *HillSlope* indicates the steepness of the curve. At *X* = *IC*_50_, the response value *Y* corresponds to the midpoint between *C*_0_ and *C_M_*, namely:

The fitting was performed using OriginPro 9.0 (OriginLab Corp., Northampton, MA, USA), and the resulting curve demonstrated a goodness-of-fit of R^2^ = 0.989. These results indicate excellent agreement between the experimental measurements and the fitted model, thereby confirming the robustness and reliability of the IC_50_ analysis.

This value was determined based on impedance-derived capacitance measurements and was found to be 3.96 μM, the standard puromycin concentration.

The equation used to calculate NIH/3T3 cell viability is presented in Equation (2).

NIH/3T3 Cell Viability (%) = (*C*T − *C*b/*C*c − *C*b) × 100(%) (2)

where *C*T is the capacitance of drug-treated cells, *C*c is the capacitance of untreated control cells, and *C*b is the background capacitance from cell-free wells.

**Figure 8:**
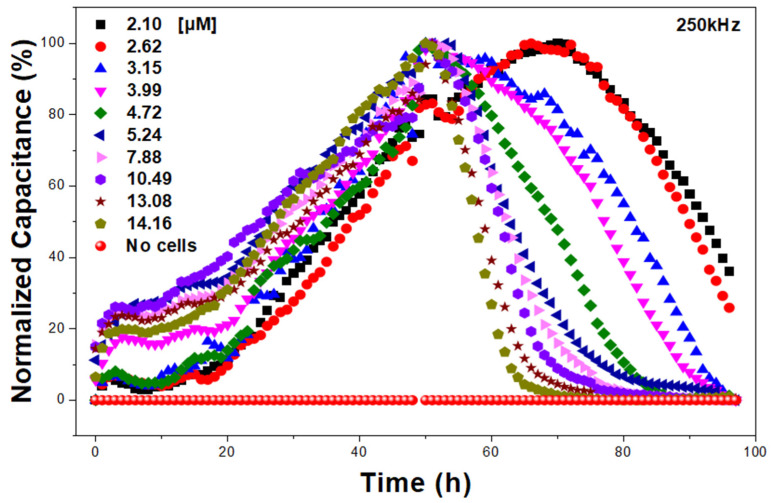
Capacitance–time data over a 0–96 h period.

Figure 8 illustrates the dose–response curve over a 96-h period, showing that cell viability decreased in a concentration-dependent manner based on capacitance-derived viability measurements. As the puromycin concentration increased, the rate of cell death accelerated, which was evidenced by a rapid decline in capacitance over time. The high reproducibility of these results further confirms the sensor’s capability for accurate drug toxicity assessment.

In our previous work, we developed a multi-well array impedance biosensor and systematically compared multiple frequencies through frequency sweep analysis and reproducibility evaluation, which identified 250 kHz as the optimal frequency for monitoring NIH/3T3 cell behavior. This frequency consistently exhibited the most stable signal-to-noise ratio and best reproducibility among the tested conditions, supporting its use in the present study [16,22].

### 3.2. Onset Time Analysis of Puromycin Cytotoxicity

The onset time, defined as the time at which significant impedance reduction is observed, was analyzed for different puromycin concentrations. As shown in Figure 8, the onset time for the 2.10, 2.62, 3.15, and 3.99 μM concentrations were 72, 68, 61, and 53 h, respectively. The results indicate that higher drug concentrations lead to faster onset times and increased cell death rates. This trend suggests a strong correlation between drug concentration and cytotoxic response, reinforcing the reliability of impedance-based biosensing for real-time toxicity assessment. The ability to distinguish onset times for different drug concentrations highlights the multi-well array impedance biosensor’s high sensitivity and reproducibility.

### 3.3. Real-Time Impedance Monitoring and Saturation Effects

In addition to IC_50_ determination, the sensor effectively captured long-term cellular responses to low-dose puromycin treatments. Notably, in concentrations below 50% cytotoxicity (IC_50_ threshold), cell death was not completed within the 96-h monitoring period. The capacitance values did not reach zero, and the impedance curves did not saturate, indicating that a fraction of the cells remained viable. These findings suggest that the multi-well array impedance biosensor provides an accurate representation of incomplete cytotoxic effects at sub-IC_50_ concentrations, distinguishing between complete and partial cell death responses. The ability to monitor these subtle variations enhances the applicability of the sensor for high-precision drug screening and biocompatibility assessments.

### 3.4. Implications for Drug Screening and Toxicity Assessment

The results of this study demonstrate that the multi-well array impedance biosensor provides a highly reproducible, label-free, real-time method for evaluating drug-induced cytotoxicity. The capacitance-based impedance measurements successfully quantified the cytotoxic effects of puromycin and provided insights into the drug concentration-dependent onset times.

The ability to detect non-saturating impedance signals at sub-IC_50_ concentrations suggests that the sensor can effectively differentiate between full and partial cytotoxic responses, making it a valuable tool for early-stage drug evaluation and toxicity profiling. Further studies incorporating additional aminonucleoside antibiotics and long-term impedance monitoring could further enhance the system’s applicability in pharmacological research and personalized medicine.

## 4. Discussion

This study demonstrated the development and application of a multi-well array impedance biosensor for the real-time, label-free monitoring of drug-induced cytotoxicity using NIH/3T3 fibroblast cells. By tracking impedance changes, particularly capacitance, as a function of time and drug concentration, the biosensor enabled the precise determination of IC_50_ values and cytotoxic onset times for puromycin. These results validate the utility of electrical impedance-based biosensing as a robust, non-invasive alternative to conventional cytotoxicity assays.

The calculated IC_50_ value of puromycin (3.96 µM) fell within the expected range previously reported in studies using colorimetric and fluorescent assays such as MTT, Trypan Blue, and Cell Counting Kit-8. The accuracy of the impedance-based IC_50_ determination has previously been validated in our group through direct comparison with conventional biological assays including MTT viability and FACS Annexin V/PI analyses. These parallel experiments demonstrated strong consistency between impedance-derived IC_50_ values and those obtained from standard biochemical assays, thereby confirming the reliability of the impedance approach. As such, the present study builds upon this validated framework and focuses on applying the impedance biosensor for quantitative drug toxicity assessment (our unpublished results, currently under review) This alignment supports the quantitative reliability of the impedance-based method and confirms its effectiveness in evaluating drug efficacy. In particular, the observed dose-dependent decrease in cell viability, along with the shortening of onset time at higher puromycin concentrations, underscores the biosensor’s high sensitivity in detecting dynamic cellular responses. In addition, although medium evaporation can be a potential confounding factor in long-term impedance measurements, our previous investigation confirmed that such effects were negligible under the present experimental conditions [22]. Therefore, the observed changes in capacitance primarily reflect cellular responses to puromycin, rather than medium-related artifacts. These findings reinforce the idea that impedance, especially capacitance, is a reliable indicator of cell status, capable of capturing early morphological and physiological changes preceding cell death.

Unlike resistance, which reflects more stable or structural changes such as cell–cell junctions and tight barrier formation, capacitance is highly sensitive to alterations in cell membrane properties, adhesion strength, and spreading area. By emphasizing the capacitance component, our approach enabled the detection of subtle changes in cellular behavior that may be overlooked by total impedance or resistance-based analysis. This is supported by previous studies advocating for component-specific impedance analysis to reveal biologically meaningful signal interpretations [13,14].

Another strength of this study lies in the integration of a multi-well array format. This design not only allows for parallel testing of multiple drug concentrations under uniform conditions, but also enhances statistical robustness by reducing the variability between wells. The reproducibility of sensor performance across different fabrication batches was confirmed in our previous work, which showed minimal inter-device variability (error margin < ±0.5%) [25,26]. Regarding stability, the biosensors were designed for single-use applications, but we verified that devices stored under sterile, dry conditions retained structural and functional integrity for up to three months. In addition, the use of semiconductor-based fabrication techniques improves reproducibility and offers scalability for large-scale or high-throughput applications. Such features are essential for screening libraries of candidate compounds during the early stages of drug development.

Despite these strengths, several limitations should be acknowledged. First, the study was limited to a single cell line (NIH/3T3) and one primary cytotoxic agent (puromycin). While puromycin’s mechanism of action is well-understood, inducing premature translation termination and cell death, it remains important to validate this biosensor’s performance across a broader range of cell types and drug classes including targeted therapies and anti-inflammatory agents. Second, the experimental window was restricted to 96 h. Prolonged impedance monitoring could reveal additional insights into chronic or delayed cytotoxic responses as well as cellular adaptation or recovery mechanisms.

Furthermore, although the impedance data were supported by live-cell imaging in this study, combining electrical measurements with molecular assays (e.g., apoptosis markers, mitochondrial potential, or transcriptomic profiling) could provide more comprehensive information on the mode of drug action. Such multimodal analysis would enhance the interpretability of impedance data and strengthen the biological significance of the observed signal changes.

In summary, this study highlights the significant potential of multi-well array ECIS biosensors for the precise, real-time evaluation of drug-induced cytotoxicity. The platform’s capacity to measure both IC_50_ values and onset times using capacitance-based analysis enables more nuanced assessments of cellular responses. As impedance biosensing technology continues to evolve, its integration with high-content screening tools and computational modeling could facilitate its application in diverse fields such as pharmacology, toxicology, regenerative medicine, and personalized drug development.

## 5. Conclusions

In this study, we developed and validated a multi-well array impedance biosensor for the real-time, label-free assessment of drug-induced cytotoxicity using NIH/3T3 fibroblast cells. The sensor successfully quantified the cytotoxic effects of puromycin through capacitance-based impedance measurements, enabling the precise determination of IC_50_ values and onset times across various drug concentrations. The IC_50_ analysis confirmed that the concentration of puromycin inducing a 50% reduction in NIH/3T3 cell viability was 3.96 µM. Real-time impedance monitoring revealed a clear correlation between puromycin dose and cytotoxic response, with higher concentrations inducing faster onset times and steeper capacitance declines. Moreover, the IC_50_ values obtained in this study provided a basis for determining the onset time of drug action, with the impedance data confirming that lower puromycin concentrations lead to progressively delayed onset times.

These findings highlight the potential of multi-well array ECIS biosensors as powerful platforms for high-precision drug screening, biocompatibility testing, and real-time toxicity profiling. Future studies involving a broader range of therapeutic compounds and extended impedance monitoring are expected to further enhance the versatility of this biosensing platform for applications in advanced drug discovery, toxicity evaluation, and precision medicine.

## Data Availability

The data that support the findings of this study are available upon request from the authors.
